# Structural insights into mechanisms underlying stabilization of a lysosomal enzyme through protein engineering

**DOI:** 10.1063/4.0000941

**Published:** 2025-10-27

**Authors:** Jennifer E Kung, Ahlam Qerqez, Tianao Yuan, Lionel Rouge, Ann Nguyen, Oliver Davis, Cathal Mahon

**Affiliations:** Denali Therapeutics, South San Francisco, CA, USA

## Abstract

Enzyme replacement therapies (ERTs) have been approved as treatments for several lysosomal storage disorders, which are characterized by deficiency of a single lysosomal enzyme leading to progressive substrate accumulation, perturbing lysosomal function and cellular homeostasis. By supplying patients with functional recombinant enzymes, these therapies have achieved clinical success but have been limited to treating peripheral symptoms due to their inability to cross the blood-brain barrier (BBB), which prevents them from addressing clinical manifestations within the central nervous system (CNS). The efficacy of ERTs is further limited by the inherent instability of the recombinant enzymes in serum. To overcome these challenges, we used a novel protein engineering strategy to mimic small molecule-induced stabilization of a lysosomal enzyme. The resulting engineered enzyme displays markedly improved serum stability relative to wild type, and fusion of the engineered enzyme to a TfR-based TransportVehicle^™^ (TV) enabled delivery of the enzyme across the BBB and increased substrate reduction in a relevant disease model. Here, we utilize structural methods, including x-ray crystallography and HDX-MS, to characterize the engineered enzyme and gain insights into the changes in its structure and conformational dynamics that lead to its enhanced stability.

